# 

**Published:** 1864-01

**Authors:** 


					JVV. ^ ?t;:''
I
(>?'

				

